# Concordance between physiotherapists and physicians for care of patients with musculoskeletal disorders presenting to the emergency department

**DOI:** 10.1186/s12873-019-0277-7

**Published:** 2019-11-10

**Authors:** E. Matifat, K. Perreault, J.-S. Roy, A. Aiken, E. Gagnon, M. Mequignon, V. Lowry, S. Décary, B. Hamelin, M. Ambrosio, N. Farley, D. Pelletier, L. Carlesso, F. Desmeules

**Affiliations:** 1 Maisonneuve-Rosemont Hospital Research Center, University of Montreal Affiliated Research Center, Montréal, Québec Canada; 2 CIUSSS Est-de-l’Île-de-Montréal, Québec, Canada; 3 Center for Interdisciplinary Research in Rehabilitation and Social Integration (CIRRIS), Québec, Québec Canada; 4 Departement of Rehabilitation, Faculty of Medicine, Laval University, Québec, Québec Canada; 5 Faculty of Health, Dalhousie University, Halifax, Canada; 6 Université de Picardie Jules Verne, Amiens, France; 7 School of Rehabilitation, Faculty of Medicine, University of Montréal, Montréal, Québec Canada; 8 CIUSSS Centre-Ouest-de-l’Île-de-Montréal, Québec, Canada; 9 Ordre Professionnel de la Physiothérapie du Québec, Montréal, Québec Canada; 10 School of Rehabilitation, Faculty of Medicine, McMaster University, Hamilton, Ontario Canada

**Keywords:** Physiotherapist, Emergency department, Primary care, Musculoskeletal disorders

## Abstract

**Background:**

Overcrowding in emergency departments (ED) is a major concern worldwide. To answer increasing health care demands, new models of care including advanced practice physiotherapists (APP) have been implemented in EDs. The purpose of this study was to assess diagnostic, treatment and discharge plan concordance between APPs and ED physicians for patients consulting to the ED for minor musculoskeletal disorders (MSKD).

**Methods:**

Patients presenting to two EDs in Montréal (Canada) with a minor MSKD were recruited and independently assessed by an APP and ED physician. Both providers had to formulate diagnosis, treatment and discharge plans. Cohen’s kappa (κ) and Prevalence and Bias Adjusted Kappas (PABAK) with associated 95%CI were calculated. Chi Square and t-tests were used to compare treatment, discharge plan modalities and patient satisfaction between providers.

**Results:**

One hundred and thirteen participants were recruited, mean age was 50.3 ± 17.4 years old and 51.3% had an atraumatic MSKD. Diagnostic inter-rater agreement between providers was very good (κ = 0.81; 95% CI: 0.72–0.90). In terms of treatment plan, APPs referred significantly more participants to physiotherapy care than ED physicians (κ = 0.27; PABAK = 0.27; 95% CI: 0.07–0.45; *p* = 0.003). There was a moderate inter-rater agreement (κ = 0.46; PABAK = 0.64; 95% CI: 0.46–0.77) for discharge plans. High patient satisfaction was reported with no significant differences between providers (*p* = 0.57).

**Conclusion:**

There was significant agreement between APPs and ED physicians in terms of diagnosis and discharge plans, but more discrepancies regarding treatment plans. These results tend to support the integration of APPs in ED settings, but further prospective evaluation of the efficiency of these types of models is warranted.

## Background

Overcrowding in emergency departments (ED) is a major concern in healthcare systems worldwide. Each year, there are almost 16 million visits made to Canadian EDs [1]. Recent reports indicate that Canada is among the countries with the longest ED waiting times. Indeed, the percentage of people waiting four hours or more in the ED is higher in Canada compared to other countries, such as Australia and the United Kingdom [2]. This situation is a major concern, especially in light of the aging population and increases in the prevalence of chronic diseases [3]. Patients presenting to EDs with minor musculoskeletal disorders (MSKD), such as tendinopathy, back pain or sprains represent more than 25% of all ED visits [4].

Several initiatives that have been implemented worldwide to reduce waiting times and improve care efficiency often include the extension of the scope of practice of non-physician health care providers [5, 6]. In the last few decades, physiotherapists have seen their scope of practice extended in various settings, such as rheumatology, orthopaedics and primary care clinics. As they are regulated health care practitioners with extensive training in this field, physiotherapists provide safe and effective care for patients with various MSKD and refer patients with conditions that are outside their scope of practice to other practitioners [4]. Often referred to as advanced practice physiotherapy (APP) models, the new roles of physiotherapists include enhancement of their responsibilities, such as: direct access to patient traditionally seen by a physician first, ability to make diagnoses, triaging surgical patients, ordering imaging or laboratory tests and prescribing certain medications [7–11]. In Australia and the United Kingdom, APPs have also been implemented in EDs and have been shown to improve access and quality of care for patients with minor MSKD [12], while providing safe and effective care and retaining high patients’ satisfaction [13–17]. The current literature on APP in EDs indicate that they can be a viable option to improve access to care for patients with MSKD [13, 16–19]. Nevertheless, only a limited amount of studies in EDs has examined the benefits and safety of these models and none have assessed diagnosis and management concordance between physiotherapists and physicians [16, 17, 19, 20]. The evaluation of the potential benefits of such models is highly context-dependent and the evaluation of these models is warranted to support further development and implementation of APP in EDs.

The objectives of the current study were to determine the diagnostic interrater reliability between ED physicians and APPs, as well as to assess treatment and discharge plan concordance, including medical imaging requests and medication recommendations, and patient satisfaction between both healthcare providers in this new model for ED patients with minor MSKD.

## Methods

### Settings

Patients were recruited from April 2017 to July 2018 from two EDs in Montréal (Canada).

### Physiotherapists and physician in the participating emergency departments

The three physiotherapists participating to this study were already working in both recruitment sites and were already involved in the ED. They had previous experience working in theses EDs as secondary contact providers, with experience ranging from 2 years to 14 years, and had experience for care of patients with MSKD, in both an inpatient and outpatient settings, with experience ranging from 2 to 29 years. Thirty-seven ED physicians participated in the current study. In the province of Québec, APP models of care include direct access to patients in hospital settings. Ability to prescribe x-rays is soon to be allowed under new regulation.

### Participants

All patients presenting to the ED and that were identified by the triage nurse as having a possible minor MSKD or identified on the ED triage list by a research assistant as having a possible minor MSKD were considered for this study on days that recruitment was taking place. Other inclusion criteria were: 1) 18 years or older; 2) legally able to consent; 3) able to understand/speak French or English; 4) resident of the province of Quebec and beneficiary of the provincial health insurance coverage (RAMQ). Exclusion criteria were patients having: 1) been previously treated by one of the APPs or physicians involved in this study; 2) an injury resulting from significant trauma (such as major motor vehicle accident) or a major musculoskeletal injury, such as open fracture or open wound; 3) obvious red flags, such as progressing neurological deficits or infection related signs or symptoms; 4) diagnosed inflammatory arthritis and 5) other active/unstable non-musculoskeletal conditions, such as pulmonary, cardiac, digestive or psychiatric conditions.

### Data collection

Prior to being seen by the APP or the ED physician, eligible participants completed a structured questionnaire with the research assistant where they provided anthropometric data as well as data on their education, employment, household income, household living status, and information on clinical variables such as the joint affected, the reason for consulting, the history of the lesion, the duration of their symptoms, the use of a walking aid and the presence of any comorbidities. Participants also completed the acute 36-item Short Form Survey Questionnaire (SF-36) to assess their health-related quality of life and, depending on the affected body region, the relevant standardized self-reported disability questionnaires among the Neck Disability Index (NDI), the Oswestry Disability Index (ODI) for spine disorders, the Disability of the Arm, Shoulder and Hand (DASH) questionnaire for upper extremity disorders or the Lower Extremity Functional Scale (LEFS) for lower extremity disorders. The SF-36 has been shown to be valid, reliable and responsive to change with various populations with MSKD [21–23]. The NDI, ODI, DASH and LEFS are standardized self-reported disability questionnaires that are validated, reliable and responsive to change [24–30].

The participants were then independently assessed by both providers. The clinical evaluation and specific physical tests used by both providers to complete the evaluation were not standardized, as so they could use any evaluation techniques or physical tests they felt appropriate to reach a diagnosis. After the assessment, the physiotherapist and the ED physician each independently completed a standardized evaluation form where they indicated the primary, and secondary if relevant, diagnoses, their request for additional medical imaging necessary to provide appropriate care or to confirm or exclude a diagnosis, their detailed treatment plan, including conservative treatment options, referral to other providers (such as orthopedics) and medication recommendations, and their discharge plan from the ED (Appendix). Due to feasibility considerations, the physiotherapist always completed the assessment before the ED physician. A research assistant ensured that the physiotherapist and the ED physician were blinded to each other’s diagnosis, treatment plan and discharge plan until both providers had completed the evaluation and had filled out the standardized form. Also, physiotherapists were asked to not initially disclose information on their diagnosis or management plan to the patients to avoid any influence on the ED physician’s diagnosis. Both providers had access to the patient’s medical file, including any previous medical imaging results available. If medical imaging was judged necessary to specify either the diagnosis or treatment plan, providers were asked to specify which type of imaging was needed. Within their treatment plans, providers were also asked to specify the relevant conservative treatment options for the patient from among the following options: 1) advice and education, 2) walking aids/orthosis, 3) home exercises or 4) specify other options. They were also asked to specify if supervised physiotherapy follow-up was relevant and any medication recommendations, either prescription or non-prescription. Regarding the ED discharge plan, providers needed to select between three options: 1) hospitalization, 2) discharge home without medical follow-up or 3) discharge with a medical follow-up (family physician, specialist as an outpatient, other). The time in minutes for each provider to complete the evaluation was recorded. To record any change in the patient’s condition that would modify the ability for the second provider to make a diagnosis, participants were asked to complete a form where they specified if their pain level had changed after the first assessment (1- no change in condition 2- pain is slightly higher; 3- pain is moderately higher; 4- pain is significantly higher). To ensure that patients’ conditions were similar for both evaluations, patients were excluded from the study if pain was recorded as significantly higher after the initial assessment by the physiotherapist.

After assessment by both the physiotherapist and the ED physician, patients completed before discharge a modified version of the 9-item Visit-Specific Satisfaction Questionnaire (VSQ-9) in relation to each provider they had seen [6, 31, 32]. Participants were informed that answers would remain confidential. The questionnaire included 7 questions rated on a 1 to 5 scale (1 = excellent and 5 = poor) and relates to satisfaction with care received, including perceived quality of assessment, personal manner of the provider and quality of advice and information received.

### Analyses

Descriptive statistics were used to describe the patients’ characteristics. Cohen’s kappa (κ) as well as Prevalence and Bias Adjusted Kappas (PABAK) with associated 95% confidence intervals (95% CI) were calculated for diagnosis concordance, medical imaging requests, treatment plan, including recommendation for medication and physiotherapy care, and discharge plans. Interpretation of inter-rater agreement was made according to the following scale in which 0–0.20 is weak, 0.21–0.40 slight agreement, 0.41–0.60 moderate agreement, 0.61–0.80 good agreement, 0.81–0.90 very good agreement and > 0.90 excellent agreement [33, 34]. Due to the multiple possible diagnoses as well as the different nomenclature sometimes used by physicians and physiotherapists, diagnoses were put into diagnostic categories based on the assessment by two independent reviewers to establish if diagnoses were concordant. The main categories were soft tissue disorders, spinal disorders, articular disorders, fracture or luxation and neurological disorders or other disorders. An independent third rater was used if consensus could not be reached. Ultimately the patient was managed according to the physician directives as the study only assessed the potential ability of the physiotherapist to manage autonomously these types of cases. In addition, Chi Square tests were used to compare treatment and discharge plan modalities and student t-tests were used to compare patient satisfaction. Analyses were preformed using SPSS version 25 (SPSS Inc., Chicago) and R version 3.4.3.

### Sample size

We calculated the sample size with an expected kappa inter-rater agreement of at least κ = 0.8, a raw proportion of agreement of 80% and power of 80% [35]. A minimum sample size of 75 participants was required, but to insure representability of the sample and to take into account the multiple MSKD encountered in an ED setting, we aimed to recruit a sample size of at least 100 participants.

### Ethics

All participants signed a consent form prior to entering the study. This study was approved by the Research Ethics Boards of both hospitals.

## Results

### Participants

**Fig. 1:**
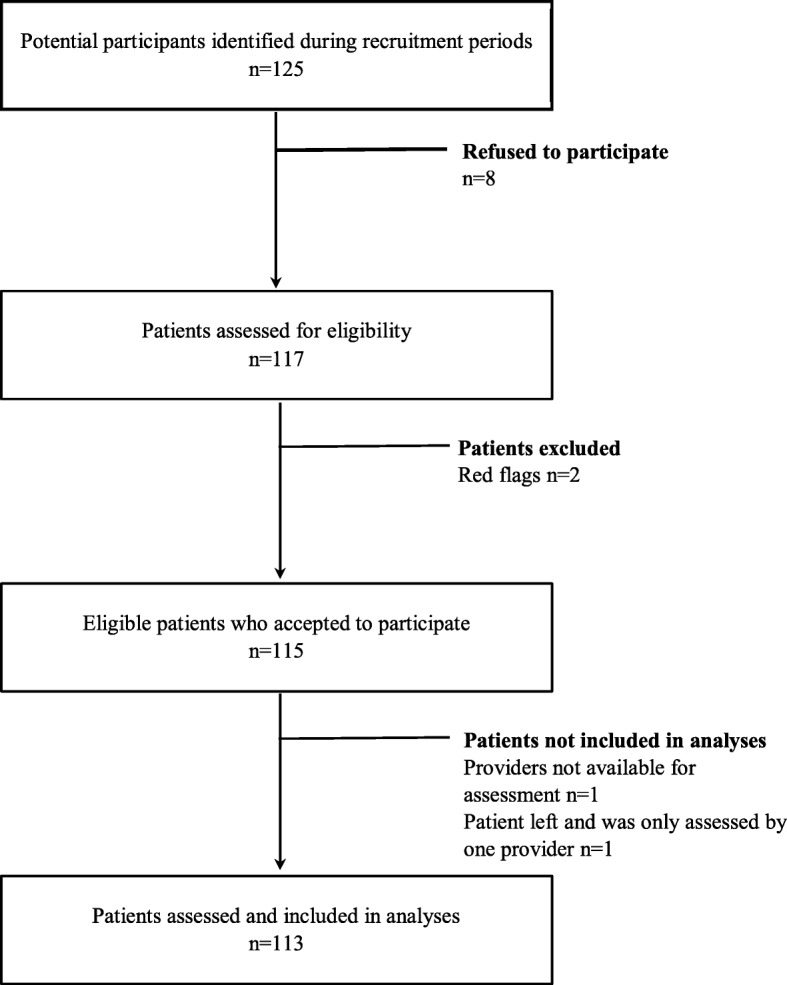
Flowchart of study participants

One hundred and twenty-five potential participants were identified after initial assessment at triage during recruitment periods, 70 in the first site and 55 in the second site. Eight declined participation to this study and the reasons were: fatigue (*n* = 1), pain was too severe (*n* = 2), was presenting to the ED to have a joint injection (*n* = 1), only wanted to see a physician (*n* = 2) and did not want to grant access to their medical file (*n* = 2). Four patients were excluded: two patients had obvious red flags (infection for both), one left after being assessed by only one out of two providers and one participant was recruited but afterward none of the providers were available for the assessments. A total of 113 participants were included in the analyses (Fig. 1).

### Subjects’ characteristics

Participants’ characteristics are included in Table 1. Subjects had a mean age of 50.3 years (SD: 17.4) with 49.6% of men and 50.4% of women. With some patients presenting disorders in more then one body region, 29% of patients had an upper limb disorder, 43% a lower limb disorder, 5% a cervical spine disorder, 4% a thoracic disorder and 19% a lumbar disorder. Most patients (51.3%) had an atraumatic disorder. Participants had a mean score of 38.3% (SD ±10.4) on the Physical Component Scale and 44.3% (SD ±13.3) on the Mental Component Scale of the acute version of the SF-36, indicating fairly impaired health-related quality of life. In terms of the various self-reported disability questionnaires based on the affected body regions, the mean scores were: LEFS 23.3 (SD ±17.3), ODI 54.8% (SD ±0.2), quick-DASH 44.2 (SD ±29.3) and NDI 25.7 (SD ±1.5).

**Table 1:** Characteristics of participants (*n* = 113)

Characteristics	n (%)	Mean (SD)
Age		50.3 (17.4)
Gender		
Male	56 (49.6)	
Female	57 (50.4)	
Education		
High school or less	50 (44.2)	
College or university	63 (55.8)	
Household income *(n = 86)*		
0–29,999$	28 (32.6)	
30,000$ - 59,999$	31 (36.0)	
60,000$ and up	27 (31.4)	
Type of injury/disorder		
Traumatic	55 (48.7)	
Atraumatic	58 (51.3)	
Using a walking aid *(n = 111)*	18 (16.2)	
Number of comorbidities per participant		1 (1.2)
36-item Short Form Survey score (%)– Acute version (SF-36) *(n = 104)*		
Physical Component		38.3 (10.4)
Mental Component		44.3 (13.3)
Lower Extremity Functional Scale (LEFS) (/80) *(n = 52)*		23.3 (17.3)
Oswestry Disability Index (ODI) (%) *(n = 22)*		54.8 (0.2)
Disabilities of the Arm, Shoulder and Hand (quick DASH) (/100) *(n = 27)*		44.2 (29.3)
Neck Disability Index (NDI) (/50) *(n* = 3*)*		25.7 (1.5)

*SD* standard deviation

### Diagnostic agreement between ED physicians and APPs and medical imaging

Overall, raw agreement regarding diagnosis between ED physicians and APPs was 87%, based on both primary and secondary diagnoses. A secondary diagnosis was present for 54% of patients (61/113). Inter-rater diagnosis agreement was very good (κ = 0.81; 95% CI: 0.72–0.90) (Table 2).

**Table 2:** Diagnostic Agreement between emergency department physicians and advanced practice physiotherapists (*n* = 113)

	Raw agreement proportion (%)	Cohen’s Kappa	95% CI	*p* value
Diagnostic Agreement	98/113 (87)	0.81	0.72 to 0.90	*p* < 0.001

In terms of requests for medical imaging, APPs and ED physicians ordered almost the same amount of imaging tests (respectively for 71% and 70% of participants, *p* = 0.884). Raw agreement proportion was 80% and inter-rater agreement was moderate (Kappa = 0.51; PABAK = 0.59; 95% CI: 0.42–0.73) (Table 3).

**Table 3:** Differences in proportion and concordance for treatment and discharge plans between advanced practice physiotherapists and ED physicians

	Recommended by MD (%)	Recommended by APP (%)	Chi-square test	*p* value	Raw agreement proportion (%)	Cohen’s Kappa	PABAK	95% CI
Medical imaging requests^□^	79/113 (70)	80/113 (71)	0.021	0.884	90/113 (80)	0.51	0.59	0.42 to 0.73
Physiotherapy care referral^§^	36/107 (34)	53/107 (50)	5.559	^*^0.018	68/107 (64)	0.27	0.27	0.07 to 0.45
Medication recommendations^¥^								
Non-prescription drugs	41/105 (39)	52/105 (50)	2.335	0.126	64/105 (61)	0.22	0.22	0.02 to 0.41
Prescription drugs	73/105 (70)	49/105 (47)	11.267	^*^0.001	69/105 (66)	0.33	0.31	0.11 to 0.49
Discharge Plan (need for medical follow-up)^‡^	78/99 (79)	78/99 (79)	0.000	1.000	81/99 (82)	0.46	0.64	0.46 to 0.77

*MD* ED physicians, *APP* advanced practice physiotherapist, ^□^*n* = 113, ^§^*n* = 107, ^¥^ *n* = 105, ^‡^*n* = 99, ^*^Significant value (*p* < 0.05)

### Treatment and discharge plans

In terms of treatment plan, APPs referred significantly more participants to physiotherapy care than ED physicians (50% compared to 34%, *p* = 0.018). Raw agreement was 64% and overall there was only slight agreement between providers (Kappa = 0.27; PABAK = 0.27; 95% CI: 0.07–0.45) (Table 3). In terms of medication, non-prescription and prescription drugs were analysed separately. APPs recommended more non-prescription drugs than ED physicians, but there was no significant difference (50% compared to 39%, *p* = 0.126). Raw agreement proportion was 61% and only slight agreement between providers was observed (Kappa = 0.22; PABAK = 0.22; 95% CI: 0.02–0.41) (Table 3). In terms of prescription drugs, ED physicians recommended them significantly more than APPs (70% compared to 47%, *p* < 0.001). Raw agreement proportion was 66% and overall only slight agreement was obtained between raters (Kappa = 0.33; PABAK = 0.31; 95% CI: 0.11–0.49) (Table 3).

In terms of discharge plans from the ED, providers were asked to specify if the patient needed any type of medical follow-up upon ED discharge (No follow-up, hospitalisation, referral to a specialist, return to general practitioner or other medical follow-up). Twenty-one percent of patients had no medical follow-up upon ED discharge, 34% were sent back to their family physician, 43% were referred to an outpatient speciality clinic and 2% were hospitalized. There was significant agreement between providers with an 82% raw agreement proportion and a moderate inter-rater agreement (Kappa = 0.46; PABAK = 0.64; 95% CI: 0.46–0.77) (Table 3).

### Evaluation length and patient satisfaction

Overall, ED physicians had significantly shorter overall consultation times (mean 5.8 min; SD ± 4.2) than APPs (mean 13.5 min; SD ±8.6) (*p* < 0.001). There were no significant differences between providers in terms of patients’ satisfaction with received care, with both providers obtaining high satisfaction scores (*p* = 0.57) (Table 4).

**Table 4:** Comparison between evaluation length and patient satisfaction for emergency department physicians and advanced practice physiotherapists

	Mean value MD (SD)	Mean value APP (SD)	Mean difference (SD)	95% CI	Student *t*-test	*p* value
Evaluation length (min)^□^	5.8 (4.2)	13.5 (8.6)	−7.7 (0.9)	−9.5 to −5.9	−8.5	< 0.001
Patient satisfaction ^§^	86.1% (18.6)	87.6% (16.3)	1.5% (2.6)	−3.6 to 6.6	0.58	0.57

*MD* emergency department physician, *APP* advanced practice physiotherapist, *SD* standard deviation

^□^*n* = 110, ^§^*n* = 91

## Discussion

Since physiotherapists and APPs are being integrated in ED teams worldwide, it is crucial to demonstrate their expertise in this role. Overall, we observed that when it comes to the care for ED patients with minor MSKD, there is significant agreement between APPs and ED physicians in terms of diagnosis and discharge plans, but there is much more variability in terms of treatment plans.

Making the right diagnosis is essential to ensure appropriate care and proper discharge plans for patients with MSKD. Our study is the first to specifically assess diagnosis, treatment and discharge concordance in the ED for patients with MSKD. Previous studies in other settings, such as outpatient orthopedic clinics, have shown equivalent ability between orthopedic surgeons and APPs in terms of diagnosis of common MSKD disorders [6, 36]. The results of our study show significant concordance between providers in terms of diagnosis. There were discrepancies between APPs and ED physicians in only 15/113 cases. It is important to specify that the ED physician’s diagnosis was considered as the reference standard, but we cannot exclude the possibility that the ED physician’s diagnosis might have been inaccurate in some cases and therefore, the lack of agreement for those 15 cases might not necessarily be due to an inaccurate diagnosis by the APPs [37, 38].

In terms of requests for medical imaging, both providers ordered about the same amount of imagery and obtained a raw agreement proportion of 80% with a moderate agreement. These numbers indicate that APPs do not order more medical imagery than ED physicians, which is similar to findings in other studies [6, 39]. The next step would be to verify the appropriateness of those imaging ordering, since this was not the main objective of this study.

In terms of treatment plans, there was divergence between both types of providers. ED physicians recommended more prescription drugs than non-prescription drugs for minor MSKD. The opposite result was found for APPs. This could reflect the fact that physiotherapists have limited prescribing rights currently in Québec and also that this topic is not covered in detail in entry level physiotherapy education. Looking more closely at the types of prescription drugs recommended by ED physicians, we found that almost one third (32%) of the patients for which a prescription drug was recommended were for opioids (results not shown). These numbers echo with the fact that Canada has already been identified as the country with the second-largest rate of opioids’ consumption worldwide [40]. Recent studies have demonstrated that opioids are usually not recommended as the first-line go to for treatment of MSKD pain, but that nonopioid medications and multimodal approaches (including physiotherapy) should be tried first [41]. There was a lot of missing information within APPs’ medication recommendations, as where they would mention a need for a prescription drug but without specifying which type, thus it is not possible to see the opioids recommendation rates with APP care in this study. This should be assessed in future studies.

We found significant differences and only slight agreement between providers for rates of referral for physiotherapy care. APPs referred significantly more to physiotherapy care than the ED physicians. Since physiotherapy care has been shown to be an effective treatment option for patients with MSKD, it is surprising that the agreement for physiotherapy care was only minimal. We believe that the fact that physiotherapy care is not covered by the national health coverage in Québec, if provided outside formal public institutions, affected the rates of referral to physiotherapy care by physicians, even more so since access to public physiotherapy has already been found to be limited [42]. ED physicians mentioned informally after the study that they often hesitate to refer to physiotherapy care since they know that a significant portion of the population does not have access to services within the public system and does not have insurance or the resources to pay for physiotherapy care in the private sector.

Another key aspect of ED practice is the discharge plan from the emergency department. Our results show that there was significant concordance between providers in terms of discharge plans from the ED. This shows that in terms of need for medical follow-up upon ED discharge, both providers tend to agree. Only two patients were identified by ED physicians as needing hospitalization for their current disorders. One presented with a suspected tibial plateau fracture with important associated disabilities and the other one a suspected pulmonary neoplasia or vascular pathology. In both cases, the physiotherapist also identified the need for a hospitalization to seek further investigations or treatments. These results show that APPs tend to recommend the same type of discharge plans compared to ED physicians, thus being able to identify the patients who require a medical follow-up upon ED discharge or need immediate medical attention.

Finally, we found no significant differences in terms of patient satisfaction with received care between providers, with both obtaining high satisfaction rates. This is similar to what was found in previous studies assessing ED physiotherapy care, with satisfaction rates being either similar with ED physiotherapy care compared to usual care or even higher with ED physiotherapy care [13, 14, 16–18].

## Strengths and limitations

This study is the first available regarding ED physiotherapy care in Canada and is also the first to assess concordance between ED physicians and physiotherapists in this setting. This study had a multicenter design with a significant number of participants (113) with various injuries and disorders, and also a large number of providers (2 EDs, 3 APPs and 37 ED physicians) which increases the generalization of our findings. The APPs already worked in the participating EDs and had extensive training and experience. Therefore, the results of this study could be different if physiotherapists with lesser training and experience were involved. Formal standardisation of required competences and training is certainly warranted if these models of care are to be expanded.

This study also had a cross-sectional design with no prospective data collection and did not include assessment of the efficacy or the efficiency of such ED models of care. The highest level of evidence for this model of care would have been obtained through a randomized controlled trial (RCT), but since this APP model of care in EDs is still emerging, we believe that our study model was warranted at this stage and is a first step to validate APP expertise in the ED. With the increased implementation of physiotherapy care models in EDs, future studies should include a prospective RCT that will allow evaluation of the efficacy as well as the cost-effectiveness of such models of care.

## Conclusion

Significant concordance in terms of diagnosis and discharge plans was found between APPs and ED physicians for patients with MSKD presenting to the ED. There were more discrepancies between healthcare providers in terms of treatment plans with APPs recommending more physiotherapy care and less prescription drugs than ED physicians. Both providers obtained high satisfaction rates. Future studies should include prospective designs, such as an RCT, and should evaluate the financial impacts, in terms of direct and indirect costs, of these type of models in ED.

Overall, these new APP models of care can be a key solution to improving access to care in Canada, especially for patients with MSKD.

## Figures and Tables

**Fig. 2:**
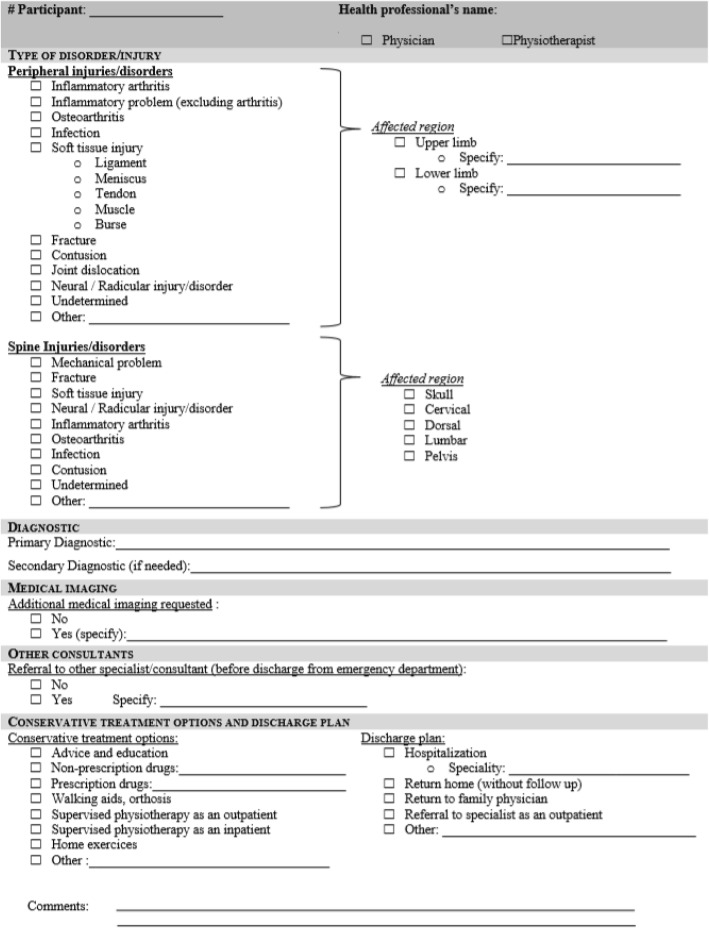
Standardized Evaluation Form

## Data Availability

Not applicable

## Abbreviations

APP: Advanced practice physiotherapy
DASH: Disability of the Arm, Shoulder and Hand
ED: Emergency department
LEFS: Lower extremity functional scale
MSKD: Musculoskeletal disorders
NDI: Neck disability index
ODI: Oswestry disability index
PABAK: Prevalence and bias adjusted kappas
RCT: Randomized controlled trial
SF-36: 36-item Short Form Survey Questionnaire
VSQ-9: 9-item Visit-Specific Satisfaction Questionnaire
κ: Cohen’s Kappa
